# Ultrasensitive quantification of TAP-dependent antigen compartmentalization in scarce primary immune cell subsets

**DOI:** 10.1038/ncomms7199

**Published:** 2015-02-06

**Authors:** Hanna Fischbach, Marius Döring, Daphne Nikles, Elisa Lehnert, Christoph Baldauf, Ulrich Kalinke, Robert Tampé

**Affiliations:** 1 Institute of Biochemistry, Biocenter, Goethe-University Frankfurt, Max-von-Laue Str. 9, 60438 Frankfurt/Main, Germany; 2 Institute for Experimental Infection Research, TWINCORE, Centre for Experimental and Clinical Infection Research, a joint venture between the Helmholtz-Centre for Infection Research and the Hannover Medical School, Feodor-Lynen Str. 7-9, 30625 Hannover, Germany; 3 Cluster of Excellence Frankfurt – Macromolecular Complexes, Goethe-University Frankfurt, Max-von-Laue Str. 9, 60438 Frankfurt/Main, Germany

## Abstract

Presentation of peptides on major histocompatibility complex class I (MHC I) is essential for the establishment and maintenance of self-tolerance, priming of antigen-specific CD8^+^ T cells and the exertion of several T-cell effector functions. Cytosolic proteasomes continuously degrade proteins into peptides, which are actively transported across the endoplasmic reticulum (ER) membrane by the transporter associated with antigen processing (TAP). In the ER lumen antigenic peptides are loaded onto MHC I, which is displayed on the cell surface. Here we describe an innovative flow cytometric approach to monitor time-resolved ER compartmentalization of antigenic peptides. This assay allows the analysis of distinct primary human immune cell subsets at reporter peptide concentrations of 1 nM. Thus, this ultrasensitive method for the first time permits quantification of TAP activity under close to physiological conditions in scarce primary cell subsets such as antigen cross-presenting dendritic cells.

## Abstract (web summary)

Antigenic peptides are loaded into major histocompatibility complex class I (MHC I) molecules after entering the ER via the TAP transporter. Here, Fischbach *et al*. develop a flow cytometry assay to monitor TAP-dependent peptide translocation that is sensitive enough to use on scarce primary cell subsets.

## Graphical abstract

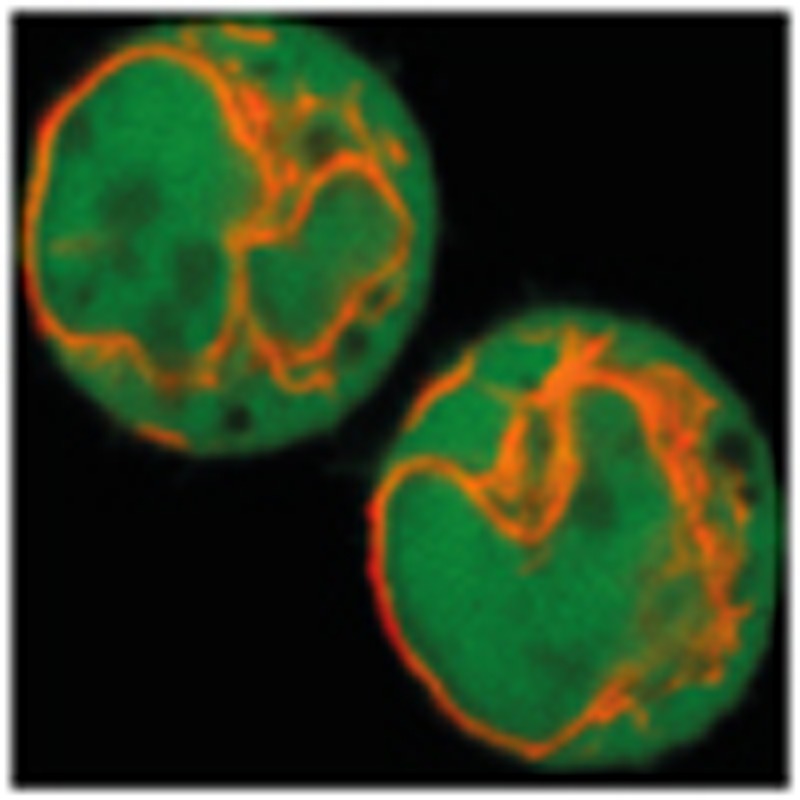

The MHC I antigen processing machinery ensures the display of the cellular proteome in form of the fragmented peptidome to CD8^+^ cytotoxic T cells (CTLs)^1^. Professional antigen-presenting cells (pAPCs), such as dendritic cells (DCs), present antigenic peptides on MHC I to prime antigen-specific CD8^+^ T cells, which then leave lymphoid organs and migrate to peripheral sites where they exert their effector functions^2^. Antigen presentation critically depends on the translocation of proteasomal degradation products from the cytosol to the endoplasmic reticulum (ER) lumen. The heterodimeric transporter associated with antigen processing (TAP), a prototype asymmetric ATP-binding cassette transporter, unidirectionally translocates peptides across the ER membrane^3^,^4^,^5^. The chaperones tapasin, ERp57 and calreticulin constitute, together with TAP and MHC I, the macromolecular peptide-loading complex^6^, which loads peptides onto MHC I molecules. On integration of a peptide into the MHC I binding groove, the chaperones are released, and the MHC I/peptide complex leaves the ER to display its antigenic cargo at the cell surface^7^. The molecular and biochemical basis of TAP function (reviewed in ref. 8) and the role of TAP as a target of viral inhibitors (reviewed in refs 9, 10) have been studied in detail. Nevertheless, previously applied *in vitro* peptide translocation assays were based on affinity purification of radiolabelled peptides trapped in the ER lumen on *N*-core glycosylation or on binding to MHC I from cell lysates and subsequent peptide quantification^11^,^12^. Also recent efforts to study TAP localization and activity still required either substantial numbers of homogenous cells, unphysiologically high concentrations of peptides or they were applied in assays with immortalized cells lines^13^,^14^,^15^,^16^. Accordingly, the role of TAP has primarily been studied in the context of target cell recognition, but not in pAPC function. This was due to the fact that antigen-translocation assays were not applicable for the study of primary cells, such as tumour cells, scarce subsets of infected cells and in particular primary antigen-presenting cells (APCs). However, the detailed knowledge on how antigen presentation is carried out in different immune cell subsets and peripheral tissue resident cells is highly desirable. Such insights are crucial for basic research, development of therapeutic approaches, as well as for vaccine development.

To study TAP-dependent peptide translocation processes in scarce primary cell types, such as immune cell subsets, we developed an ultrasensitive method that allows high-throughput, time-resolved and multiplexed flow cytometric analysis of the ER compartmentalization of antigenic peptides. Our analysis reveals that distinct immune cell subsets exhibit different magnitudes of TAP-dependent peptide translocation with monocytes and DCs showing the highest antigen-translocation efficacy amongst the different human peripheral blood mononuclear cell (PBMC) subsets.

## Results

### FACS-based assessment of antigen compartmentalization

**Figure 1:**
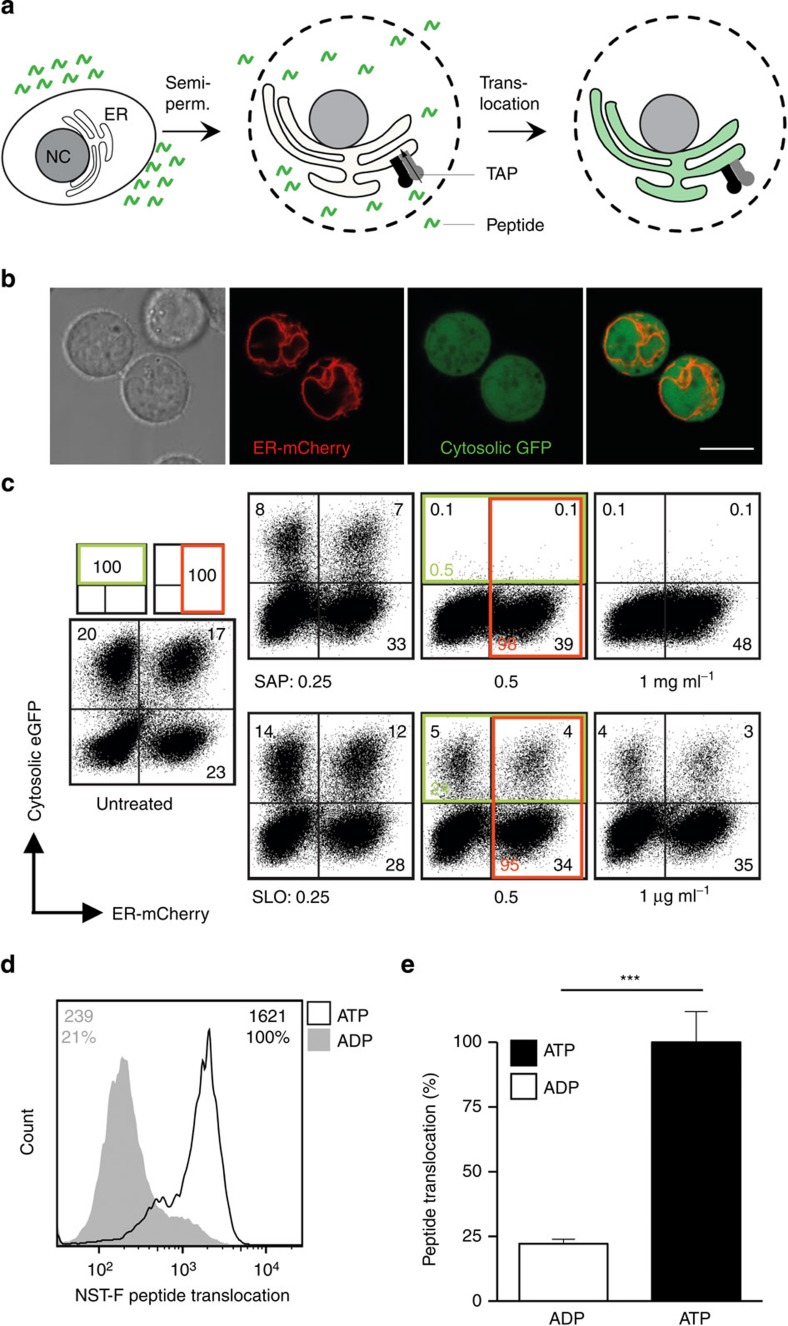
Flow cytometric assay monitoring antigen translocation. (**a**) Schematic workflow of the TAP-dependent peptide translocation (TPT) assay. The human B-lymphoblastic cell line Raji was semipermeabilized with saponin (SAP) or streptolysin O (SLO) and fluorescent peptide (1–10 nM of NST-F) was added. Peptide translocation was carried out at 37 °C for 15 min in the presence of ATP or ADP (10 mM each). To stop the translocation process, an excess of EDTA (20 mM) was added and cells were analysed by flow cytometry. NC, nucleus; ER, endoplasmic reticulum; TAP, transporter associated with antigen processing. (**b**) Confocal microscopy of cytosolic eGFP expression and ER-resident mCherry expression in stably cotransduced Raji cells. Scale bar represents 10 μm. (**c**) Raji cells were cotransduced with lentiviral vectors coding for cytosolic eGFP and ER-resident mCherry. After semipermeabilization with different concentrations of SAP (0.25, 0.5 and 1 mg ml^−1^) or SLO (0.25, 0.5 and 1 μg ml^−1^), cells were incubated at 37 °C for 15 min and analysed by flow cytometry. The fluorescence intensities were measured using 530-nm and 640-nm (long-pass) channels for GFP and mCherry, respectively. (**d**) The histogram overlay of the single-cell events (530-nm channel) demonstrates an ATP-dependent compartmentalization of antigenic epitopes (black line: ATP; grey filled: ADP; numbers indicated mean fluorescence intensity (MFI; absolute and per cent of ATP)). (**e**) The MFI of the fluorescein channel for ATP (black bar) versus ADP (white bar) samples is shown as relative translocation rate (%). The error bars indicate the s.d. of 15 independent experiments (Wilcoxon test, ***P≤0.0002, *n*=12).

Arguing that semipermeabilized cells loaded with fluorescence labelled peptide would allow TAP-dependent peptide translocation (TPT) monitoring by flow cytometry (Fig. 1a), we used the human histone H3-derived reporter epitope RRYQNSTC^(F)^L (NST-F) labelled with fluorescein. Since (poly)peptides are subjected to co- and post-translational modifications within the ER, including *N*-core glycosylation, ER-resident NST-F is glycosylated and will not be targeted into the ER-associated degradation pathway followed by retrotranslocation^17^. As the TPT assay relied on intact ER membranes, two different permeabilization protocols involving either saponin (SAP) or streptolysin O (SLO) were tested with stably transduced Raji cells expressing cytosolic eGFP and ER-resident mCherry (Fig. 1b). Upon SAP incubation, these cultures showed significantly decreased eGFP fluorescence. The average of 0.5% remaining eGFP-expressing cells was calculated by comparing 20% (eGFP^+^) plus 17% (eGFP^+^mCherry^+^) eGFP-expressing cells in the untreated cell cultures (left panel in Fig. 1c) with eGFP-expressing percentages of 0.1% (eGFP^+^) plus 0.1% (eGFP^+^mCherry^+^) in SAP-treated cell cultures (Fig. 1c, middle panel upper row). At the same time mCherry fluorescence remained at ~98%, as calculated by comparing 17% (eGFP^+^mCherry^+^) plus 23% (mCherry^+^) mCherry-expressing cells in the untreated cell cultures with mCherry-expressing percentages of 0.1% plus 39% in SAP-treated cell cultures. The equivalent calculations for percentages of remaining fluorescent populations on SLO treatment revealed that the eGFP-expressing population was reduced to ~24%, whereas mCherry-expressing cells remained at 95% compared with untreated samples (Fig. 1c, middle panel lower row). Collectively, these results indicated that SAP and SLO treatment perforated the plasma membrane more efficiently than the ER membrane, whereas this bias was even more pronounced in SAP-treated cells. To next study intracellular peptide translocation, Raji cells were SAP-treated and incubated with NST-F. Only in the presence of ATP, but not ADP, the cells showed significant increase of the NST-F-mediated fluorescence (Fig. 1d,e).

### Real-time analysis of antigen translocation

**Figure 2:**
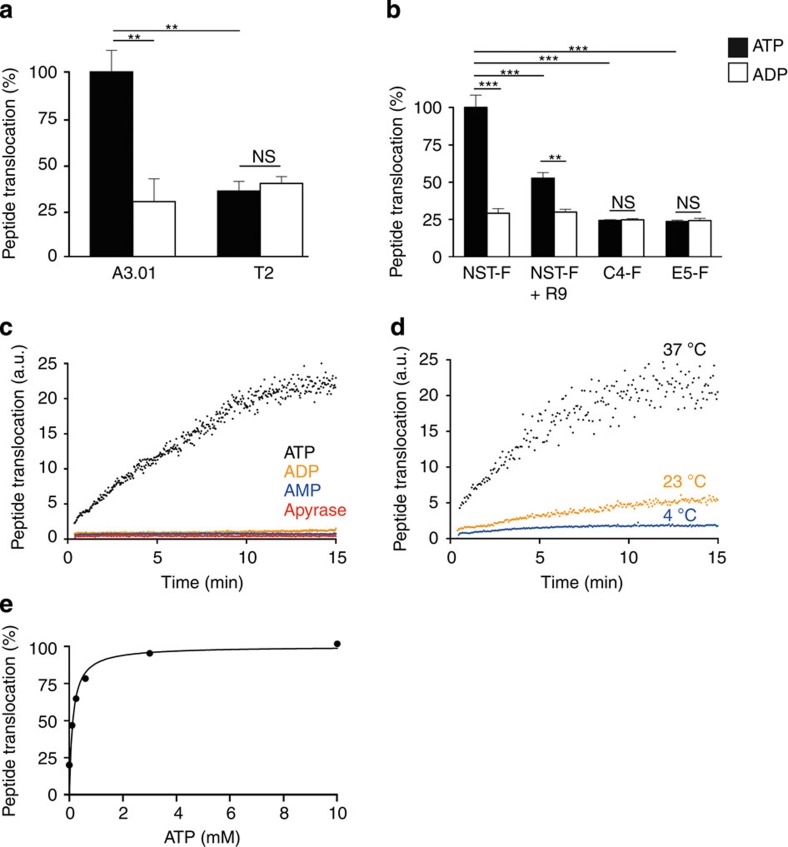
TAP dependence of ER antigen compartmentalization. (**a**) Human TAP-proficient (A3.01) and TAP-deficient (T2) T lymphocytes were semipermeabilized and TPT assay was performed. Values were normalized to the peptide translocation rate of A3.01. The error bars indicate the s.d. (Mann–Whitney *U*-test; **P≤0.0076, *n*=6). (**b**) The TPT assay was carried out in Raji cells in the absence or presence of 2 μM of the high-affinity epitope R9, a fluorescently labelled epitope lacking the *N*-core glycosylation site (C4-F) or the non-binder peptide E5 (E5-F), respectively. The detected mean fluorescence intensities (530-nm channel) were normalized to the NST-F/ATP sample. The error bars indicate the s.d. (Mann–Whitney *U*-test; **P≤0.0011; ***P≤0.0002, *n*≥6). (**c**) The mean fluorescence intensity (MFI) of the 530-nm channel was analysed for 15 min at 37 °C with a frequency of 0.3 Hz in the presence of ATP, ADP, AMP (10 mM each) or apyrase (0.1 units per 2 × 10^5^ cells). (**d**) The assay was carried out at 37 °C, 23 °C and 4 °C as well as analysed for 15 min at 37 °C with a frequency of 0.3 Hz. (**e**) The TPT assay was carried out using 10 nM of reporter peptide NST-F and increasing concentrations of ATP. Translocation rates relative to the ADP control values are plotted versus ATP concentrations.

To further verify the TAP dependence of the above-detected peptide translocation, intracellular antigen translocation was analysed in the TAP-deficient lymphocyte cell line T2. Whereas the TAP-proficient cell line A3.01 revealed a fluorescent signal, the TAP-deficient T2 cells did not (Fig. 2a). On coincubation of semipermeabilized Raji cells with NST-F together with unlabelled high-affinity peptide RRYQKSTEL (R9) the fluorescence signal was reduced (Fig. 2b), presumably because of high-affinity peptide R9 competing with NST-F for TAP binding. To verify that the accumulation of fluorescence signal was glycosylation dependent, the TPT assay was next performed with the RRYC^(F)^KSTEL peptide (C4-F) devoid of an *N*-core glycosylation site. Due to rapid retrotranslocation of non-glycosylated peptides^18^, no enhanced fluorescent signal was detected (Fig. 2b). However, comparison of ATP and ADP samples revealed a constant peptide translocation background of ~25% (Figs 2b and 1e), which can be assigned to unspecific binding of fluorescently labelled peptides to semipermeabilized cells, as indicated by a similar background staining of the TPT assay performed with the EPGYC^(F)^NSTD (E5-F), which is refractory to TAP binding and thus is not transported (Fig. 2b). In addition, similar background levels were detected in TAP-deficient T2 cells (Fig. 2a). The analysis of monocytes in control experiments with R9 and C4-F peptides as well as with increasing ATP and ICP47 (a viral TAP inhibitor) concentrations revealed overall moderately reduced background levels when compared with Raji cells (Supplementary Fig. 1a).

For time-resolved flow cytometry, NST-F peptide and ATP were added to semipermeabilized Raji cells and the measurement was initiated with a time lapse of 20 s. Although a significantly enhanced fluorescence was detected in the presence of ATP, controls with AMP, ADP or ATP together with the ATP hydrolyzing enzyme apyrase, did not show increased fluorescence (Fig. 2c). Compared with incubation at 37 °C, a decreased peptide translocation was found at 23 °C, whereas translocation activity was basically undetectable at 4 °C (Fig. 2d). Translocation activities at different ATP concentrations revealed a *K*_m_ value of 0.13±0.05 mM ATP (Fig. 2e), which was in the range of *K*_m_ values described previously^19^.

### Viral inhibition of antigen translocation

**Figure 3:**
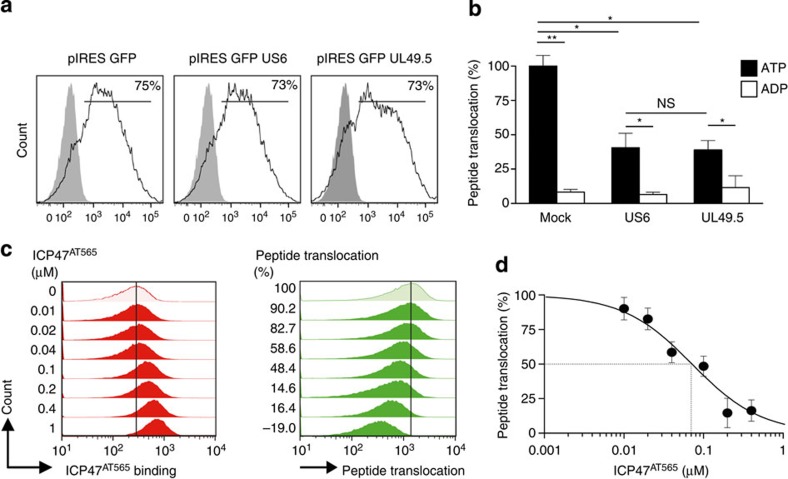
TAP-dependent peptide translocation is inhibited by virus-derived TAP specific inhibitors US6, UL49.5 and ICP47. (**a**) Raji cells were transiently nucleofected with plasmids encoding the inhibitors US6 and UL49.5. Equal expression was confirmed by bicistronic expression of GFP. Transfection with the empty IRES-GFP vector served as control (mock). (**b**) Peptide translocation of nucleofected Raji cells was analysed by the respective mean fluorescence intensities of the 530-nm channel, which were normalized to the ATP sample of the mock control. The error bars indicate the s.d. (Mann–Whitney *U*-test; *P≤0.05; **P≤0.004, *n*≥6). (**c**) Simultaneous dual flow cytometric detection of peptide translocation in the 530-nm channel (right panel) and ICP47^AT565^ binding in the 488/575-nm channel (left panel) was carried out for increasing concentrations of ICP47^AT565^ in Raji cells. Numbers indicate the concentration of ICP47^AT565^ in μM (left panel) and the mean per cent of transport (right panel). As a control, peptide translocation was carried out in presence of DMSO. (**d**) The mean fluorescence intensities detected in the 530-nm channel were ADP background subtracted and plotted against the increasing ICP47^AT565^ concentrations to determine the IC_50_ value (65 nM with a 95% confidence interval from 36 to 115 nM) of ICP47^AT565^ binding (dotted line) in Raji cells. The error bars indicate the s.d.

We next investigated Raji cells transiently nucleofected with the human cytomegalovirus-derived membrane glycoprotein US6 (ref. 20) or the bovine herpesvirus 1 protein UL49.5 (ref. 21) (Fig. 3a). Of note, on semipermeabilization recombinantly expressed US6 and UL49.5 remained integrated into the peptide-loading complex, while vector-derived intracellular GFP was washed out (as indicated in Fig. 1b), thus avoiding interference with fluorescently labelled NST-F. In ~70% of the transduced Raji cells peptide translocation was quantitatively inhibited, whereas the remaining 30% untransduced cells presumably contributed to the residual TAP activity (compare Fig. 3a and Fig. 3b). Furthermore, flow cytometric analysis of TAP binding by site-specifically ATTO565-labelled ICP47 (ICP47^AT565^) in parallel with NST-F translocation revealed a dose-dependent inhibition of TAP-mediated peptide translocation (Fig. 3c), resulting in an IC_50_ value for ICP47^AT565^ of 65 nM with a 95% confidence interval from 36 to 115 nM (Fig. 3d). Of note, this value determined by our method is in a similar range as IC_50_ values previously determined by classical methods^22^. We further confirmed the TAP dependence of antigen translocation in primary human monocytes. Performing the TPT assay in the presence of increasing amounts of ATP revealed a *K*_m_ value of 0.15±0.04 mM ATP for TAP-dependent peptide translocation in primary human monocytes (Supplementary Fig. 1b). The high sensitivity of our assay even with primary cells is underlined by detection of NST-F translocation with 1 nM peptide (Supplementary Fig. 1c). For isolated monocytes an IC_50_ value for ICP47^AT565^ of 13 nM with a 95% confidence interval from 9 to 18 nM was determined (Supplementary Fig. 1d and Fig. 1e).

### Antigen translocation in primary human immune cells

**Figure 4:**
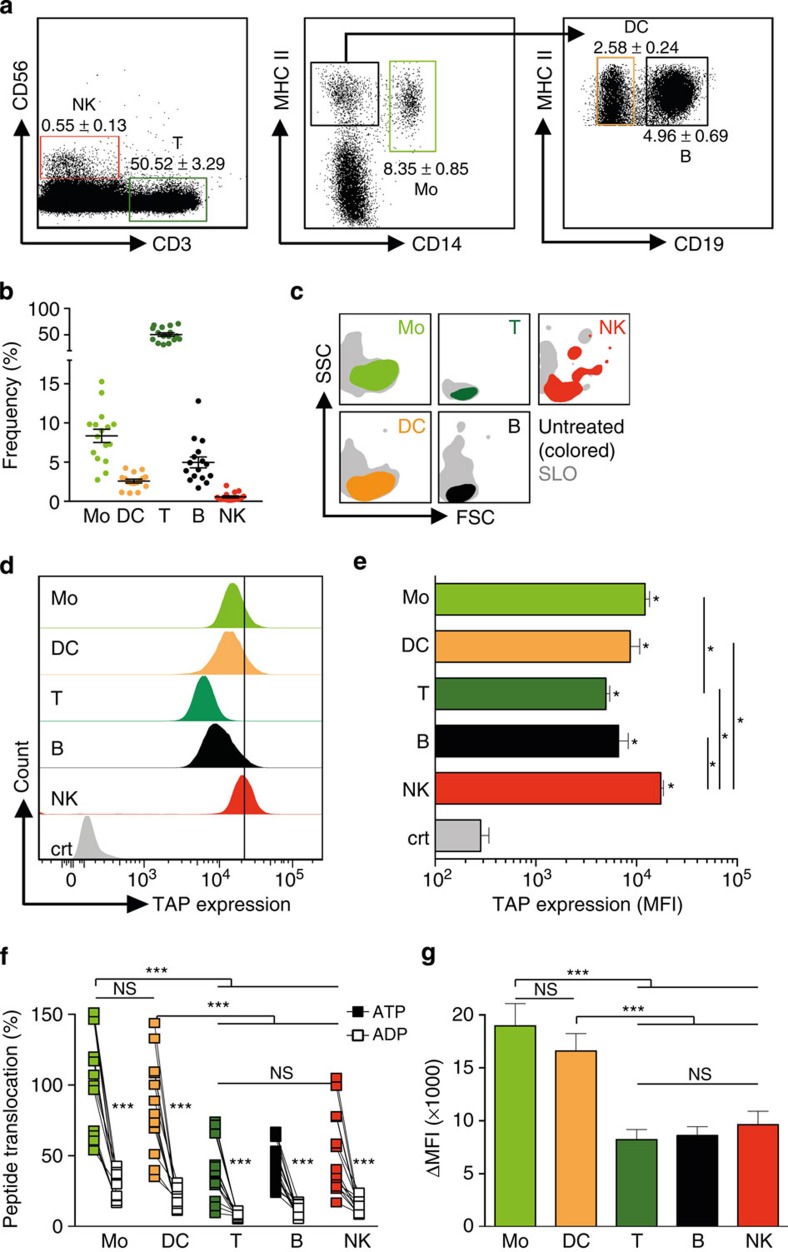
Antigen compartmentalization in peripheral mononuclear blood cells. (**a**) Flow cytometric analysis of PBMC. Human PBMC were stained with fluorophore-coupled antibodies specific for CD3, CD14, CD19, CD56 and MHC II. NK cells (NK), T cells (T), monocytes (Mo), dendritic cells (DC) and B cells (B) were separated as indicated for one representative donor. (**b**) Frequencies of selected subsets in PBMC as detected by flow cytometry. The error bars indicate the s.e.m. (**c**) Forward and side scatter depiction of PBMC subsets before and after treatment with SLO. (**d**) TAP expression levels of PBMC subsets were determined by intracellular TAP staining. (**e**) Statistical analysis of TAP expression in PBMC subsets. The error bars indicate the s.e.m. Asterisks indicate significant differences in the expression levels of TAP and were calculated using Mann–Whitney *U*-test (*P≤0.0296, *n*=4). (**f**) PBMC were semipermeabilized with SLO and incubated with 300 nM NST-F in the presence of 10 mM ATP or ADP. All cell subsets were analysed separately for their TAP-dependent peptide translocation activity in comparison with the respective ADP sample. Translocation rates for all subsets were normalized to translocation rates of monocytes. The error bars indicate the s.e.m. Statistical analysis was performed using the Kruskal–Wallis test (***P≤0.0001, *n*=14). (**g**) The mean fluorescence intensities detected in the 530-nm channel of ADP controls were subtracted from the mean fluorescence intensities of ATP samples (ΔMFI). This was done for each ADP/ATP pair of each cell type from all experiments performed (***P≤0.0001, *n*=45). The error bars indicate the s.e.m. Statistical analysis was performed using a repeated measures one-way ANOVA.

Finally, ER compartmentalization was monitored in distinct PBMC subsets, including CD3^−^CD56^+^ natural killer (NK) cells, CD3^+^CD56^−^ T cells, CD14^−^CD19^+^MHC II^+^ B cells, CD14^−^CD19^−^MHC II^+^ DCs and CD14^+^MHC II^+^ monocytes. First cell subsets were stained and then the TPT assay was performed (Fig. 4a). In PBMC derived from 14 individual donors, the frequencies for NK cells (0.55±0.13%), DC (2.58±0.24%) and T cells (50.50±3.29%), as well as monocytes (8.35±0.85%) and B cells (4.96±0.69%) were overall rather consistent (Fig. 4b). On SLO treatment of primary immune cells, FSC/SSC properties of the cells differed, indicating that their shape and granularity were altered (Fig. 4c). All tested PBMC subsets expressed TAP1 (Fig. 4d). Whereas lymphocytes, including T cells and B cells, showed similar TAP1 expression, NK cells, monocytes and DCs showed significantly enhanced TAP1 expression (Fig. 4e). Interestingly, independent of the detected TAP1 levels, our TPT assay revealed that monocytes and DCs displayed significantly higher TAP-dependent peptide translocation than NK cells, T and B lymphocytes (Fig. 4f). The net TAP activity of the cell subsets further demonstrated that independent of the varying background levels detected in individual donors, TAP-dependent peptide translocation of monocytes and DCs was significantly higher than that of lymphocytes and NK cells (Fig. 4g).

## Discussion

Here we describe a novel approach to address key questions on how the ER compartmentalization of antigens is regulated in scarce cell subsets, and what role TAP-dependent peptide translocation plays during viral infection and tumour development. Both SAP and SLO treatment effectively semipermeabilized the cell membrane of Raji cells, while their ER membrane was affected only marginally. Nevertheless, compared with SAP, the milder SLO protocol turned out to be more suitable for the treatment of more sensitive cells such as PBMC. The biochemical functions of TAP as determined by our assay were consistent with earlier results obtained in classical assays^12^. Time-resolved flow cytometry confirmed the impact of the incubation temperature on TAP activity^16^ and revealed maximal peptide accumulation within ~15 min incubation time. Notably, the reporter peptide concentration of 1–10 nM used in our flow cytometric TPT assay was several orders of magnitude lower than the concentration of radiolabelled peptide typically used in classical *in vitro* assays (4.5 (14) to 0.25 μM (ref. 22)), highlighting that in our assay rather physiological conditions, reflecting the short-lived peptidome, are tested. Despite background levels of peptide transport differed slightly in Raji cells and isolated primary monocytes, our TPT assay yielded highly reproducible results with the different cells tested, highlighting the robustness of our assay. As demonstrated by competitive inhibition, our TPT assay allows the analysis of TAP-dependent translocation of many different antigenic peptides as indicated in Fig. 2 and Supplementary Fig. 1. The previously described TAP inhibitors US6, UL49.5 and ICP47 significantly reduced TAP-dependent peptide translocation. Thus, it will be of particular interest to examine TAP activity of primary cells infected with HCMV or other viruses that evade antigen presentation by inhibiting TAP. All PBMC subsets we analysed showed TAP-dependent peptide translocation. Notably, monocytes and DCs showed higher TAP1 expression than lymphocytes and exhibited overall enhanced TAP translocation activity, whereas in NK cells despite of TAP1 expression similar to DCs only moderate TAP translocation activity was detected. Interestingly, monocytes showed similar TAP-dependent peptide translocation activity as DCs. Remarkably, TAP activities of the different immune cell subsets were highly reproducible for cells derived from single donors, whereas the absolute TAP activity values of different donors showed some variation (Fig. 4f). Nevertheless, calculation of the net TAP activity for single-cell subsets revealed a high comparability between donors, suggesting that the TAP expression levels are not indicative of TAP activity (Fig. 4g). In lymph nodes, DCs can prime naive antigen-specific T cells. Accordingly, we expected to detect significant TAP activity in this subset, whereas the high TAP activity of monocytes was surprising, considering their limited CTL stimulation capacity as pAPC precursors^23^. Their high TAP activity might explain why monocytes can serve as an antigen reservoir during chronic infection^24^. In addition, it will be interesting to assess the TAP activity of the different subsets within the CD14^−^CD19^−^MHC II^+^ population. The capacity of subsets specialized in antigen presentation might be higher than in other DC subsets. Two explanations can be offered why APCs and lymphocytes show different TAP activities: either (i) glycosylation was very efficient in APC subsets, but not in lymphocytes and NK cells or (ii) the TAP-dependent peptide translocation was influenced by additional cofactors expressed in monocytes and APC, but not lymphocytes and NK cells. Because glycosylation is a prerequisite for the viability of cells, and the majority of proteins are post-translationally modified by glycosylation^25^, the second conclusion is more favourable. Thus, we suggest that the bottleneck of peptide accumulation within the ER is represented by the translocation of antigenic peptides catalysed by TAP. Further studies on TAP activity in primary human immune cells are needed to elucidate the exact conditions of differential TAP activity in selected cell subsets. Nonetheless, the method presented here now allows for analyses of selected subsets of primary human DCs, which will provide additional information together with the classical cellular assays applied so far^26^.

In conclusion, our TPT assay allowed for the first time monitoring of TAP-dependent reporter peptide translocation in scarce primary human immune cell subsets. Further analysis with different cell types, including newly identified subsets of human DCs and macrophages, will help to elucidate mechanisms underlying antigen presentation in humans. For the study of pathogenesis of infectious diseases as well as malignancies, this method will provide crucial additional information needed for the development of new vaccination strategies and novel drugs. Since high-throughput screens with small compound libraries depend on reliable, quick and simultaneous detection of multiple parameters such as peptide translocation, inhibitor binding and lineage marker expression, the FACS-based translocation assay is ideally suited, because it enables both, multiplex formats and very fast readout. These key advantages and in particular the minute number of cells needed for our assay will allow new studies in the field of antigen presentation.

## Methods

### Cells, plasmids and materials

Human Raji B cells (ATCC CCL86)^27^, human T cells A3.01 (ref. 28), and T2 cells (ATCC CRL1992)^29^ were analysed. HEK293T cells were used for transient transfection experiments. Peptides NST-F (RRYQNSTC^(F)^L, cysteine labelled with fluorescein)^30^, C4-F (RRYC^(F)^KSTEL), R9 (RRYQKSTEL), E9 (EPGYTNSTD) and E5-F (EPGYC^(F)^NSTD) were synthesized by solid-phase Fmoc chemistry and purified by reversed phase C_18_ HPLC^31^. NST-F and C4-F were labelled with iodoacetamido fluorescein followed by reverse phased purification. The identity of the peptides was verified by MALDI-MS. Peptide concentrations were determined by a micro bicinchoninic acid assay or a photometric detection via the fluorescent dye. The plasmids pUS6-IRES-eGFP and pUL49.5-IRES-GFP were cloned in pIRES-eGFP (Clontech) and applied by nucleofection. The expression of the viral proteins was indirectly monitored by flow cytometry^32^. For lentiviral transduction, a three-vector system was utilized. The transfer plasmid encoding for the ER-targeted mCherry variant was cloned from pViFCGdBH-eGFP by fusion of mCherry to the KDEL retention peptide at the C terminus. Together with the packaging plasmid SgpΔ2 and envelope plasmid pMD.G2, pViFCGdBH-mCherry-KDEL was used for transient transfection of HEK293T cells^33^.

### Viral inhibitor ICP47

Full-length ICP47 with N-terminal His-tag and C-terminal Strep II-tag was constructed using the specific oligonucleotides: 5′- AGTCACATATGCATCA CCATCACCATCACCATCACCATCACGGAGGCTCAATGTCGTGGGCCCTGG -3′ and 5′- AGCTACTCGAGTCATTTTTCGAACTGCGGGTGGCTCCAAGCGCTACGGTTAC CGGATTAC -3′. Amplified fragments were cut with NdeI and XhoI restriction enzymes and inserted into the pET20B(+) expression plasmid (Invitrogen). The single cysteine ICP47 mutant H45C was generated using the oligonucleotide 5′- GAACCGCCGTGTGCGACCCGGAGC -3′. The sequences were confirmed by DNA sequencing. ICP47-H45C plasmids were transformed into the BL21pLysS strain for expression and purification from inclusion bodies by use of Ni-NTA agarose. After elution and dialysis (cut-off 4–6 kDa, ZelluTrans, Roth), the protein was labelled with ATTO565-maleimide (AT565; Atto-Tec, Siegen) for 5 h at 25 °C using a fivefold molar excess. ICP47^AT565^ was separated by loading onto a streptactin Sepharose and eluted with 5 mM of D-desthiobiotin (Sigma). The fluorescently labelled protein was dialysed against water and lyophilized. The identity of ICP47 labelled with ATTO565 was confirmed by MALDI-MS. For transport studies, ICP47^AT565^ was dissolved in DMSO.

### Lentiviral transduction

The three plasmid-encoded lentiviral vectors were generated by calcium chloride transfection of HEK293T cells by using the pViFCGdBH plasmid (transfer plasmid) either containing eGFP or mCherry-KDEL in combination with SgpΔ2 (packaging plasmid) and pMD.G2 (envelope plasmid)^33^. After 48 and 72 h, supernatants were collected, filtered through a 0.45-μm filter and concentrated by centrifugation on a sucrose layer for 2 h at 116,000 *g*. The pellets were resuspended in RPMI medium (Gibco) and stored at −80 °C. Raji cells were seeded in RPMI supplemented with 10% FBS (Biochrom) at a density of 2 × 10^5^ in a 24-well plate. After 24 h, vector particles were added and cells were centrifuged at 200 *g* for 1 h. Medium was exchanged after 2-h incubation at 37 °C. Five days post transduction, cells were analysed by flow cytometry (FACS Aria, BD or Attune, Applied Biosystems and LSRII sorb BD). Fluorescence was detected using 530/30 and 640LP default filters with corresponding filter ranges of 515/45 and >640 after excitation with the blue laser at 488 nm for eGFP and mCherry-KDEL, respectively. Samples were analysed using the FlowJo 7.6.5 software.

### Semipermeabilization of cells

2 × 10^5^ Raji cells were semipermeabilized using different concentrations of saponin (0.25, 0.5 or 1 mg ml^−1^; Sigma) or streptolysin O (0.25, 0.5 or 1 μg ml^−1^; Abcam). Permeabilization with SAP was performed for 25–40 s at RT and cells were pelleted afterwards. For permeabilization with SLO, cells were incubated at 4 °C with SLO for 15 min and then washed to remove residual SLO. TPT assay was performed when cells were semipermeabilized at least to 70%. After 15 min at 37 °C, cells were analysed by flow cytometry in phosphate-buffered saline (PBS) buffer supplemented with 10 mM of MgCl_2_. Fluorescence properties were detected as described above for eGFP and mCherry-KDEL. Samples were analysed using the FlowJo 7.6.5 software.

### Peptide translocation

Raji cells (2 × 10^5^) were semipermeabilized using either 0.25 mg ml^−1^ of saponin or 0.25 μg ml^−1^ SLO. Transport was carried out in the presence of 10 mM ATP/ADP, 10 nM NST-F in PBS buffer supplemented with 10 mM of MgCl_2_ for 15 min at 37 °C in 50 μl. The reaction was stopped by addition of 150 μl PBS supplemented with EDTA (20 mM). Samples were analysed using FlowJo 7.6.5 software reporting the mean fluorescence intensity . In case that real-time measurement was performed, cells were analysed by flow cytometry (Aria, BD; 502/530 ex/em) directly after adding nucleotide and peptide. Samples were analysed using FlowJo 7.6.5 software reporting the mean fluorescence intensity.

### Nucleofection

Raji cells (2 × 10^6^) were transiently nucleofected using the Lonza Nucleofection system (Solution V). 2 μg of pUS6-IRES-GFP and pUL49.5-IRES-GFP expression plasmids were purified by use of the GeneClean purification kit (Qbiogene) and pure plasmids were used as described in the optimized protocol for Raji transfection (Lonza). Empty pIRES-GFP (Clontech) was used as a control. 24 h post nucleofection, cells were collected and analysed for GFP expression and peptide transport by flow cytometry as described above.

### Primary blood cells

Human PBMCs were isolated from buffy coats using Ficoll gradient centrifugation (Biocoll, Biochrom AG). CD14-positive monocytes were enriched by MACS selection using CD14 MicroBeads (Miltenyi). For FACS staining of PBMC Fcγ receptors were blocked by the addition of 10% Gamunex of the final volume. PBMCs (2 × 10^6^ to 5 × 10^6^) were stained with anti-CD3-PerCP (UCHT1), anti-CD14-APC (M5E2), anti-CD19-PE (HIB19), anti-CD56-Pacific blue (HCP56), anti-HLA-DR-APC-Cy7 (L243; Biolegend/BD Biosciences) for 15 min at 4 °C. After surface staining, up to 5 × 10^6^ PBMCs were semipermeabilized with 0.5 μg ml^−1^ SLO. Peptide transport was performed and analysed as described above. TAP expression was monitored by the anti-TAP1 monoclonal antibody mAb148.3 produced in the laboratory. BD Cytofix/Cytoperm Fixation/Permeabilization Solution Kit was used according to the manufacturer’s instructions after surface staining to detect TAP expression in primary human immune cell subsets.

### Statistical analysis

All statistical analyses were performed with GraphPad Prism6. Only non-parametric tests such as Mann–Whitney *U* (unpaired) or Wilcoxon (paired) were used. For grouped analysis, the corresponding Kruskal–Wallis ANOVA (unpaired) was used, which includes a Dunn’s correction for multiple comparisons.

## Author contributions

D.N., H.F. and M.D. designed and performed the experiments with Raji cells. H.F. and M.D. designed and performed the experiments with primary human immune cells and transduced Raji cells, analysed the results and prepared the figures. M.D., H.F., U.K. and R.T. prepared the manuscript. U.K. and R.T. conceived the ideas and directed the work.

## Additional information

**How to cite this article:** Fischbach, H. *et al*. Ultrasensitive quantification of TAP-dependent antigen compartmentalization in scarce primary immune cell subsets. *Nat. Commun.* 6:6199 doi: 10.1038/ncomms7199 (2015).

## Supplementary Material

Supplementary Information Supplementary Figure 1
