# Supplementary material for: Ultrasensitive quantification of TAP-dependent antigen compartmentalization in scarce primary immune cell subsets
Source: Nat Commun. 2015 Feb 6;6:6199. doi: 10.1038/ncomms7199 (PMC4347055; doi:10.1038/ncomms7199)
Supplement: Supplementary Information — Supplementary Figure 1 [file ncomms7199-s1.pdf]

Suppl. Fig. 1

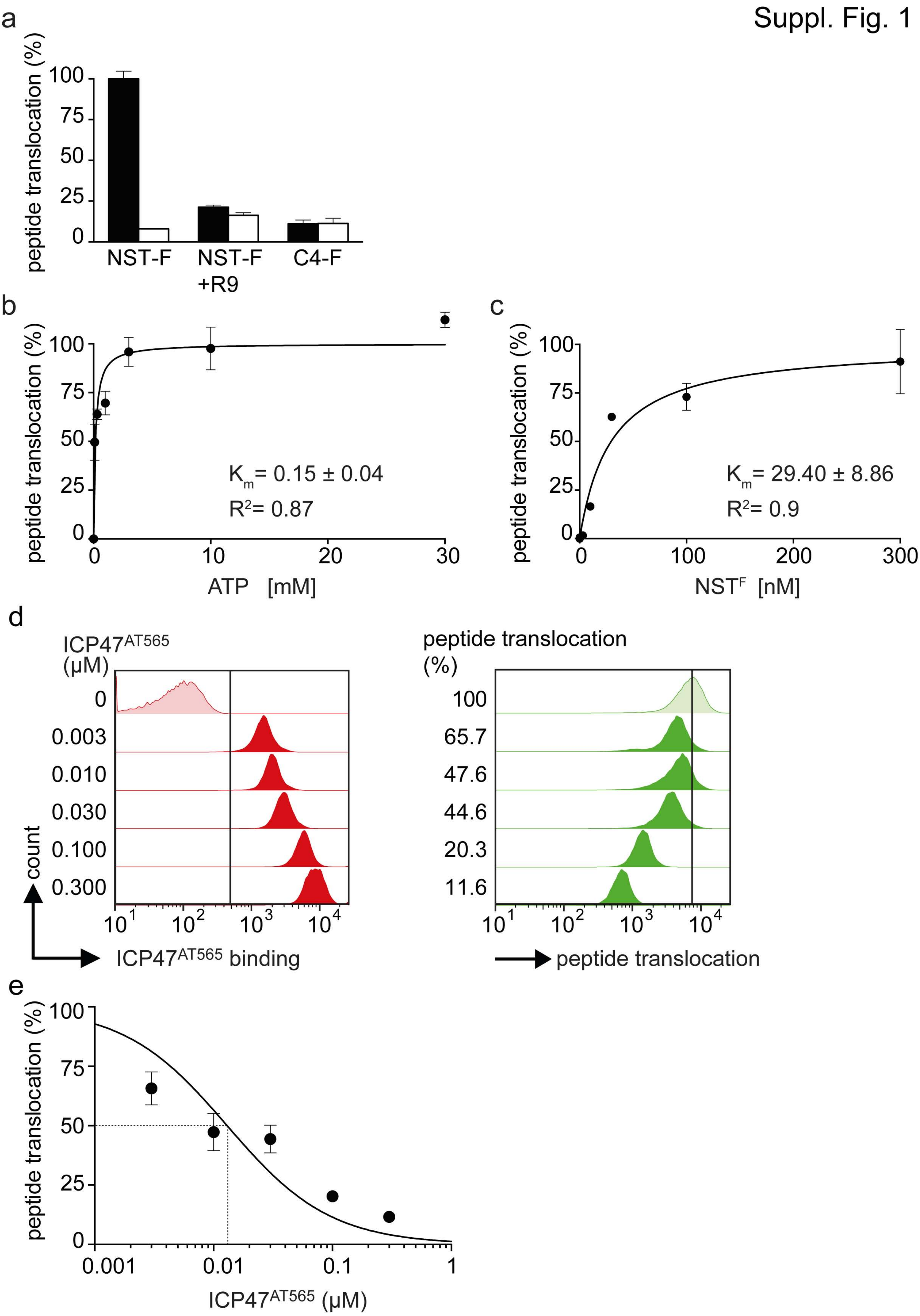

Supplementary Figure 1 | Parameters of TAP activity in monocytes are reminiscent of those in Raji cells. (a) Flow cytometry-based translocation assay was carried out in monocytes in the absence or presence of 2 µM of the high-affinity epitope R9 and a fluorescently labeled epitope lacking the N-core glycosylation site (C4-F). The detected mean fluorescence intensities (530-nm channel) were normalized to the NST-F/ATP sample. The error bars indicate the standard error of mean (n ≥ 4). (b) The TPT assay was carried out using 10 nM of reporter peptide NST-F and increasing concentrations of ATP. Translocation rates relative to the ADP control values are plotted versus ATP concentrations (n = 2). (c) The TPT assay was carried out using 10 mM ATP and increasing concentrations of NST-F peptide. Translocation rates relative to the ADP control values are plotted versus NST-F concentrations (n = 2). (d) Simultaneous dual flow cytometric detection of peptide translocation in the 530-nm channel (right panel) and ICP47^{AT565} binding in the 488/575-nm channel (left panel) was carried out for increasing concentrations of ICP47^{AT565} in monocytes. Numbers indicate the concentration of ICP47^{AT565} in µM (left panel) and the mean percent of transport (right panel). As a control, peptide translocation was carried out in presence of DMSO. (e) The mean fluorescence intensities detected in the 530-nm channel were ADP background subtracted and plotted against the increasing ICP47^{AT565} concentrations in order to determine the IC50 value (13 nM with a 95% confidence interval from 9 nM to 18 nM) of ICP47AT565 binding (dotted line) in monocytes. The error bars indicate the standard error of mean (n = 2).
